# Laser-Based Lighting: Experimental Analysis and Perspectives

**DOI:** 10.3390/ma10101166

**Published:** 2017-10-11

**Authors:** Nicola Trivellin, Maksym Yushchenko, Matteo Buffolo, Carlo De Santi, Matteo Meneghini, Gaudenzio Meneghesso, Enrico Zanoni

**Affiliations:** DEI—Department of Information Engineering—University of Padova, Via Gradenigo 6B, 35131 Padova, Italy; maksym.yushchenko@dei.unipd.it (M.Y.); matteo.buffolo@dei.unipd.it (M.B.); carlo.desanti@dei.unipd.it (C.D.S.); menego@dei.unipd.it (M.M.); gauss@dei.unipd.it (G.M.); zanoni@dei.unipd.it (E.Z.)

**Keywords:** laser diode, lighting, photoluminescence, remote phosphors

## Abstract

This paper presents an extensive analysis of the operating principles, theoretical background, advantages and limitations of laser-based lighting systems. In the first part of the paper we discuss the main advantages and issues of laser-based lighting, and present a comparison with conventional LED-lighting technology. In the second part of the paper, we present original experimental data on the stability and reliability of phosphor layers for laser lighting, based on high light-intensity and high-temperature degradation tests. In the third part of the paper (for the first time) we present a detailed comparison between three different solutions for laser lighting, based on (i) transmissive phosphor layers; (ii) a reflective/angled phosphor layer; and (iii) a parabolic reflector, by discussing the advantages and drawbacks of each approach. The results presented within this paper can be used as a guideline for the development of advanced lighting systems based on laser diodes.

## 1. Foreword

In recent years several research groups and lighting manufacturers have been working toward the development of higher efficacy light sources, with higher luminous performance and improved optical management, aiming at possible ways for new applications.

Over the last fifteen years, gallium nitride (GaN)-based light emitting diodes (LEDs) have been demonstrated to be excellent light sources, and—thanks to the intensive research effort—they have changed from an early technology (in the late 90s) to a mass product with record efficacies in excess of 300 lm/W [1]. Nowadays GaN-based LED technology is widely used for homes, cars, streets, and many other applications.

GaN-based LEDs emit monochromatic violet, blue or green radiation depending on the alloy composition; the use of phosphorescent materials (based on garnets, aluminate or silicate doped with rare earths), allows the conversion of the short-wavelength radiation emitted by the LEDs into a broad yellow–green spectrum, thus permitting the generation of white light.

While a relevant research effort is still being carried out to increase the performance of GaN LEDs, new research fields are being explored, with the aim of solving some of the technological limitations of LED lighting.

One of these fields is solid state laser lighting, which is potentially showing several advantages over LED technology. The basic idea is to create white light sources based on GaN-based laser diodes, instead of using the conventional GaN LEDs.

The reason the use of a semiconductor laser might be interesting for general lighting is not the stimulated coherent emission typical of a laser, but the high light intensity, luminous efficiency and optical management of the light beam.

Despite the high potential of laser-based lighting, no quantitative and systematic study of the advantages and issues of this approach has been published to date in the literature. The aim of this paper is to describe the issues related to the development of laser-based white light sources. The first part of the paper presents a critical comparison between laser lighting and conventional LED lighting; the second part of the paper reports experimental data on the characterization, stability and reliability of phosphors for laser lighting, which were submitted to high temperature/light intensity stress. In the last part of the paper, we compare the performance of three systems for laser lighting, which were developed within this study. The three systems are based respectively on transmissive phosphor layers, reflective/angled phosphor layer, and on the use of a parabolic reflector.

## 2. Introduction

This section critically analyzes the advantages of laser lighting with respect to conventional LED-based lighting by describing how the use of lasers impacts the étendue, efficiency droop and phosphor performance/reliability.

### 2.1. Reduced Source Étendue

Solid state lasers offer an optical advantage over standard LED solutions, thanks to the possibility of using ultra-narrow optics. The size of an optical element is related to the size of the light source. This is mainly related to the étendue of an optical system; the étendue can be simply described as the product of a source emitting area and the solid angle of the emitted beam. The étendue is critical in optical design, since it never decreases if the optical power is conserved. This translates into the fact that collimating an LED with an emitting area of 1 mm^2^ and 120° aperture, for example, into a 5° beam implies that the equivalent area of the source increases accordingly, and so does the area of the optical element (lens or reflector). Since the emitting area of a laser diode is much smaller than that of an LED (in the orders of tens of square microns vs. square millimeters, for the same optical power) it is possible to design more efficient optics and solutions able to achieve smaller divergence angles. These solutions are particularly interesting in the automotive and projection industries.

### 2.2. Efficiency Droop

A solid-state laser may also offer an advantage in terms of efficiency, since Laser Diodes (LDs) do not suffer from the well-known efficiency droop affecting LEDs. The efficiency droop (or simply just droop) is the decrease in internal efficiency as the injected current increases, a phenomenon typical for LEDs and generally ascribed to Auger recombination [2,3] or carrier spill-over [4,5]. The main drawback of efficiency droop is the reduction of the luminous flux achievable from a single LED for a given chip area. Stimulated emission, compared to spontaneous emission, is a significantly faster process and both Auger recombination and carrier spillover are less evident. Since carrier recombination proceeds mainly through stimulated emission, once LDs are biased above their threshold they do not suffer from detectable droop. The high temperature behavior of the devices is limited by thermal rollover and series resistance, which still pose a limit to the maximum driving current. It becomes quite clear therefore that, since LED droop is a physical phenomenon that can be reduced [6] but not completely eliminated, solid-state lighting (SSL) based on laser may become of much greater interest in the near future when the LED luminous efficiency will reach its peak.

### 2.3. Phosphors for LED Lighting: Operation and Issues

General lighting systems produce white light; from monochromatic sources (like LEDs and laser diodes, LD) white light can be obtained through the red-green-blue (RGB) approach, or via phosphor conversion. Due to higher color rendering performance, global efficiency and thermal stability, phosphor conversion is the most used method in the lighting industry. White light is therefore generated starting from a blue light source, typically with a peak wavelength of 440–460 nm, which is then partially converted into a broader emission coming from the phosphors. The conversion mechanism has two different types of energy losses: (a) Stokes shift, the difference between the energy of the absorbed and emitted photons (approximately 81% efficiency at typical wavelength); (b) the photoluminescence quantum yield is lower than one (typically 90% [7] for the yttrium-aluminum-garnet (YAG) type of phosphors used in this work). The energy losses also depend on temperature and irradiance levels since both thermal quenching and saturation are key parameters in phosphor luminescence. In laser activated remote phosphor (LARP) systems the excitation energy density can be much higher than LED lighting; therefore, saturation becomes particularly critical when the phosphor luminescence decay time is not sufficiently short to convert the excited absorbed electrons. For this work, phosphors with fast decay time (70/80 ns for YAG:Ce^3+^) were chosen, thus allowing the possibility to neglect the saturation phenomena. 

It should be noted that, since the phosphors are encapsulated into a transparent medium, with a generally low thermal conductivity, they suffer from localized heat and therefore the phosphor operating temperature is dependent on the incoming absorbed irradiance and their global efficiency. In LED-based devices, phosphors can be either: (i) placed directly over the LED chip; (ii) encapsulated into the silicone lens of the LED or (iii) located at a certain distance from the monochromatic LED source (solution also known as remote phosphor technology).

A critical aspect in solid state laser lighting consists of the greater irradiance on the phosphor material, with respect to LED-based white light configuration. Studying and understanding the phosphor properties and the effects of subsequent localized heat is fundamental for the final application. The aim of this section is to investigate both reversible and irreversible thermal driven phosphor efficiency decreases, in particular thermal quenching and thermal phosphor reliability. 

### 2.4. Characterization and Reliability of Lasers and Phosphors in LARP Systems

For the above-mentioned reasons, phosphor properties and reliability are primary issues for the development of laser-based lighting sources. In the following we summarize the main scientific results on the topic that have been reported so far in the literature.

The YAG:Ce^3+^ phosphor conversion efficiency and quantum efficiency are in the range of 70% and 87%, respectively [8], at room temperature. Temperature, however, has a strong effect on both efficiency and reliability and has to be carefully considered while studying LARP systems. YAG:Ce^3+^ thermal quenching has been found to be dependent on the Ce concentration, whereas the intrinsic quenching temperature is quite high (above 400 °C) [9], but becomes a limiting factor in useful concentrations for operating temperatures above 250 °C. Considering the adverse effect of temperature, remote phosphors (RP) have been presented by several authors as a possibility to reduce phosphor temperature and improve phosphor efficiency [10]. 

For GaN-based LD, ageing induces an increase in the threshold current (Ith), whereas slope efficiency is generally not affected [11,12]; this mechanism is attributed to an increase in non-radiative recombination within the active region of the devices. Operating temperature also plays a role in the degradation, with a minor impact with respect to the driving current [13,14]. However, it should be noted that the reliability of GaN heterostructures has achieved satisfactory results in recent years, and the continuous development of GaN for GaN technology also ensures the stability of LD structures.

Phosphor degradation also represents a possible threat to solid state laser lighting. In these particular applications, wavelength conversion is mostly implemented through remote phosphors (placed far away from the laser diode); this approach is often called laser activated remote phosphor (LARP). Several scientific results [15] indicate that the phosphors in LEDs are subject to a gradual reduction in conversion efficiency and a change of correlated color temperature as a consequence of thermal stress. It has been shown that the degradation rate is strongly correlated with the stress temperature with an activation energy of 1.36 eV for a time to failure (TTF) of 70% [16].

In particular it is reported that, during normal operation, phosphors can show significant self-heating and, as a consequence, the conversion efficiency of the phosphors decreases. Exposure to long-term stress at moderate/high temperature levels can lead to remarkable degradation of the phosphors or the phosphor binder [15]. Degradation mainly consists in a decrease in conversion efficiency and in a worsening of the chromatic properties of the light-emitting diode-phosphor system. Operating phosphors at higher temperatures will induce faster degradation kinetics related to the chemical degradation of the encapsulating medium (polycarbonate-, epoxy- or silicone-based materials are often used for LEDs). 

With regard to remote phosphors for LD lighting, a higher operating temperature should be achieved, caused by the much higher irradiance reaching the phosphor surface. For this reason, conventional silicone-based matrixes are not suitable for lighting, and more effective deposition methods must be studied/implemented.

### 2.5. Example Applications

Laser lighting has already found commercial application in niche markets, mainly in automotive lighting [17] and digital projection [18,19]. 

With regard to the first, LARP systems are able to deliver narrower high beams (resulting in a longer penetration distance), which can be managed by means of smaller optical elements, which can be easily integrated into adaptive optical systems.

The projection industry is also developing and implementing LARP-based light sources for high illuminance digital projectors; phosphors might be both used as monochromatic converters (for example to create bright green spots) if the blue radiation is completely absorbed from the phosphor itself, or as a conventional white light source if some of the blue LD radiation passes through the phosphor layer unconverted. The main advantages of the use of laser for the projection is: (i) efficiency; (ii) size; (iii) color saturation and (iv) lifetime. To achieve the desired lifetime, several strategies are implemented; the aim is to reduce the operating temperature of the conversion phosphor layers. The most-used solution sees phosphors placed on the outer border of a spinning wheel, thus spreading the irradiated energy into a much larger area. It is easy to see that, although these solutions are suitable for projection applications, they are too complex for the general lighting market.

### 2.6. Transmission vs. Reflection

The LARP system can be divided into two main categories based on the conversion structure: (i) phosphors deposited on a transparent substrate; and (ii) phosphors deposited on a reflective substrate. 

The transmission conversion structure has a simpler mechanical construction, and an optical advantage, since the radiation converted by the phosphors can be controlled by means of both a lens and a reflector. The drawback of the transmission structure is a high optical loss, since the phosphor emission is also backscattered toward the laser source.

In the reflected conversion structure, phosphors are placed on a reflecting substrate, thus all the emitted light is reflected toward the same hemisphere of the incident beam. Besides reaching a higher efficiency, the thermal management of the phosphors is simplified since one side of the reflector can be placed on a heatsink or thermally conductive substrate.

## 3. Phosphor Characterization and Stability Tests

For any light source, to achieve a high color rendering and a specific color temperature, a rich and void-free emission spectrum in the 400–800 nm visible wavelength region is required; this condition requires that in phosphor converted light sources, blue emitters such as LEDs or lasers need to be coupled with a specific mixture of phosphors, emitting in the yellow, green and red region. In this section we report on the reliability test carried out on yttrium aluminum garnet (Y_3_Al_5_O_12_) phosphors doped with cerium^3+^ (YAG:Ce^3+^). The YAG phosphor has a yellow–green luminescence with an emission peak of 550 nm, and as such this phosphor is required to be mixed with a red light source, such as nitride-based phosphors or red/amber LEDs to achieve a high color rendering output. Nevertheless, YAGs are some of the most commonly used phosphors in SSL, thus the authors believe that the reported tests are particularly interesting for laser applications, allowing the estimation of reversible and non-reversible thermal efficiency decreases. An understanding of the thermal quenching behavior is also important to allow the design of high efficiency systems, as good reliability is fundamental to guaranteeing that the LARP solution is robust enough to be used in the general lighting market. 

### 3.1. Experimental Details

The YAG:Ce^3+^ used in this section are commercially available phosphors in powder form, they need to be deposited onto a substrate in order to be excited in the proper way. The substrates need to be stable over temperature, optically transparent or reflective (according to the adopted configuration).

The phosphors used in this study have a peak emission of 550 nm, the chromatic diagram coordinates x = 0.426 and y = 0.548 and an average particle size of 8.5 µm. The typical excitation wavelength is 450 nm. 

Phosphors were deposited by drop casting on a sapphire substrate with a diameter of 12.7 mm, and a thickness of 3 mm. Sapphire was chosen to guarantee high temperature stability and good thermal conductivity. Ethyl alcohol and benzyl alcohol were used as solvents to allow the spin coating of the phosphor-diluted powder. 

Ethyl alcohol is a standard non-denaturated laboratory-grade pure alcohol, whereas benzyl alcohol (80708 from Fluka, Merck KGaA, Darmstadt, Germany) is 99.9% pure. The phosphor was treated at 250 °C for 6 min in order to evaporate the alcohol carrier as much as possible. By using the described procedure, it was possible to obtain a fairly uniform, thin phosphor layer with enough mechanical stability to allow management and installation in the optical LARP system.

The photoluminescence (PL) properties of drop-casted YAG phosphor samples were measured as a function of the temperature and laser irradiance, whereas reliability tests were carried out as function of both temperature (pure high temperature storage tests) and localized laser irradiance.

For the optical power stress tests, a commercial high-power blue laser diode was used to excite the phosphor layer. The laser diode is a commercial device based on a gallium nitride (GaN) compound semiconductor with a peak emission wavelength of 450 nm, and a maximum optical power in excess of 1.5 W at a current of 1.5 A. The LD was packaged in a TO-56 can package. 

The photoluminescence lifetime measurements were carried out by means of an ILX LightWave (Newport Corporation, Irvine, CA, USA) current pulser driving the LD with 1 µs pulses for a 1 ms period. A wideband (200–1100 nm) 150 MHz high speed Si photodiode and a 1 GHz oscilloscope were used to collect the data; to reduce the signal from the laser diode, a long pass filter was placed in front of the photodiode. For the phosphor thermal properties, the setup was based on a controlled thermostatic chamber.

In the experiment, the LD emits an optical power (OP) of 1.1 W, with a spot size on the sample of 0.412 mm^2^, the power density being 2.667 W/mm^2^. The experiment was repeated, reducing the LD OP to 526 mW, with a spot size on the sample of 0.394 mm^2^; the power density was 1.335 W/mm^2^. The experiment was directed toward the stability study of the samples prepared by means of two different phosphor solvents, ethanol and benzyl alcohol.

The first sample prepared with benzyl alcohol was subjected to several hours of test under 2.667 W/mm^2^ optical power. Based on the test results two other samples, each prepared with different solvents, were subjected to a test at a reduced irradiance of 1.335 W/mm^2^.

**Figure 1:**
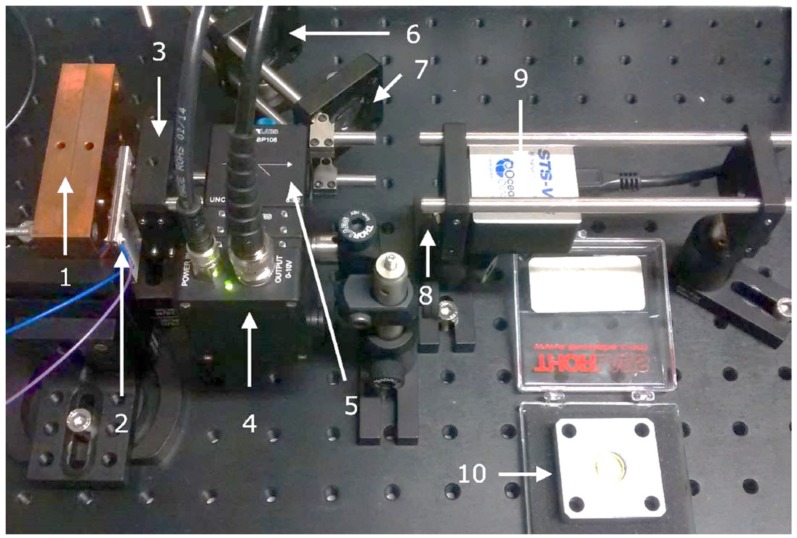
LED-based photoluminescence measurement setup.

For the thermal reliability tests, a specific optical set-up was designed to perform the photoluminescence measurement of the phosphor layer. The light source was a temperature-controlled ((1) in Figure 1) 450 nm LED (2), the light beam was collimated by means of an aspherical lens (3), and then sectioned and measured by means of a beam splitter 8:92 (4) and a high sensitivity photodiode (5). The photoluminescence radiation emitted from the phosphor (8 and 10) was then acquired by means of two Ocean Optics STS-VIS spectrometers (Ocean Optics Inc., Largo, FL, USA), one for the reflected (7 and 6) and the other for the transmitted spectrum (9). It is therefore possible to measure the excitation intensity and the converted absolute spectrum, as well as the variation in the spectral emission of the conversion layer.

### 3.2. Results

**Figure 2:**
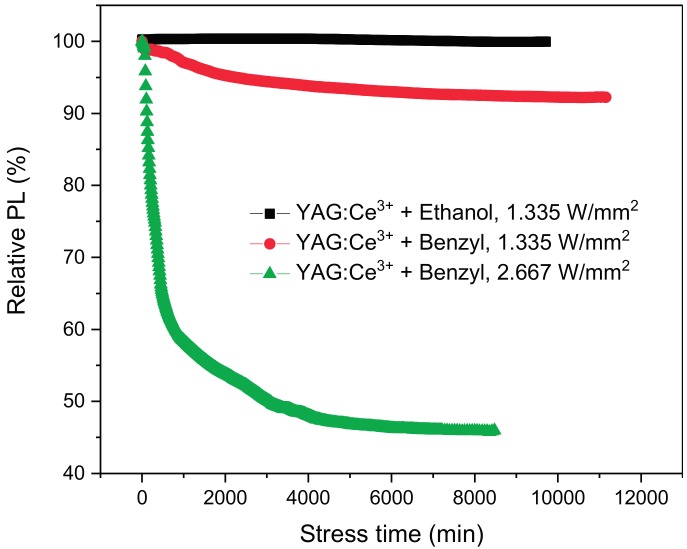
Degradation at constant optical power stress, comparison of ethanol and benzyl alcohol solvents.

The results from the optical power stress tests are reported in Figure 2. It is interesting to notice that the phosphor deposited by means of ethanol after 8000 min of operation shows good stability, whereas the same amount of phosphor deposited by means of benzyl alcohol subjected to 1.335 W/mm^2^ and 2.667 W/mm^2^, respectively, show an 8% and 55% PL decrease after the same amount of time.

**Figure 3:**
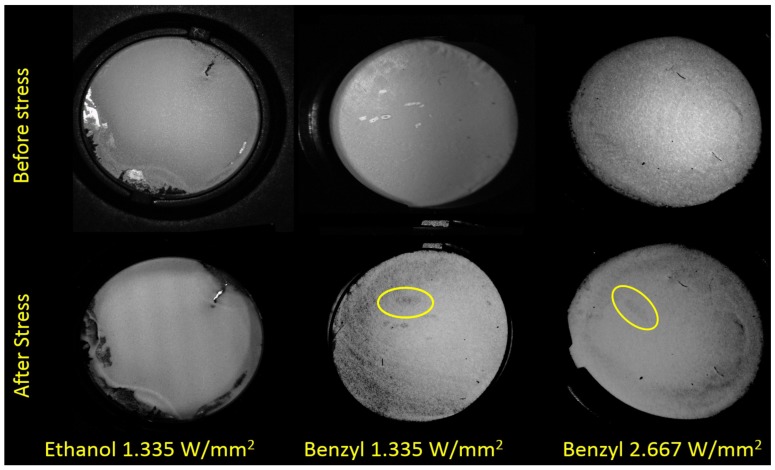
High contrast photoluminescence images of the phosphor templates deposited by means of benzyl and ethanol solvents, before and after the ageing treatment.

Photoluminescence images of the phosphor samples were acquired before and after the photodegradation tests. The images in Figure 3 present the phosphor photoluminescence (post filtered to remove the blue radiation and to improve the contrast) when excited by a 450 nm LED light source; the effects of localized photo-degradation are visible (highlighted by circles in Figure 3) on different samples tested at various excitation levels. It can be observed that only on the benzyl-based deposition samples a clear variation in emission was detected, whereas the ethanol-based deposition was not affected. Phosphor observed under white light does not show any burning of the phosphor region. The cause of the degradation in the benzyl-deposited phosphors is currently not clear, the main hypothesis is that it is related to an incomplete evaporation of the residual solvents, further studies will be carried to clarify the results. In order to avoid such effects, an ethanol solvent was used in this study.

#### 3.2.1. Characterization and Thermal Quenching Results

**Figure 4:**
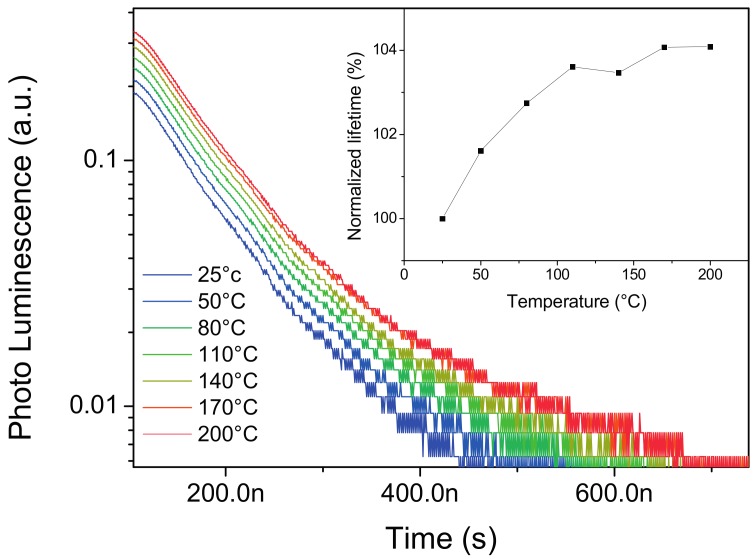
Time-resolved photoluminescence signal of the YAG:Ce at different temperatures. Inset: normalized luminescence lifetime as a function of temperature.

In Figure 4, we report on the photoluminescence lifetime measurement performed on the YAG:Ce^3+^ phosphor sample as a function of temperature; the lifetime has a time constant of 72 ns at 25 °C, which gradually increases with temperature. The lifetime time constant has been found to increase by about 4% from 25 °C to 200 °C. This value and behavior is compatible with reports from previous studies [20], the absence of variation in the decay constant at time zero also suggests that no trap is present or has a visible effect on the analyzed temperature region.

**Figure 5:**
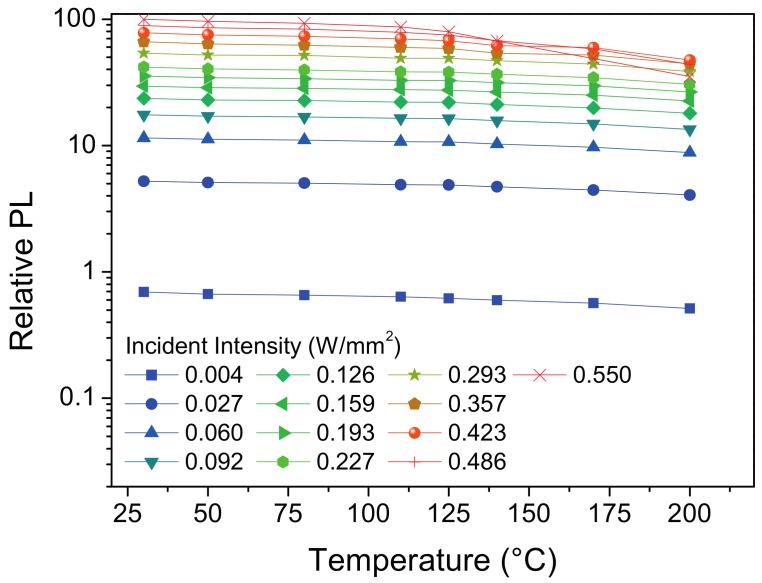
Semilog plot of the relative photoluminescence signal of the YAG:Ce phosphor as a function of carrier temperature and at different incident intensities.

The results from the photoluminescence as a function of incident irradiance and carrier temperature are presented in Figure 5. The relative PL is normalized to the maximum current value at 30 °C; when increasing the carrier temperature, first a gradual decrease in efficiency occurs, followed by a sudden drop indicating thermal quenching. Thermal quenching is only visible for higher incident irradiance since the combined effect of the carrier temperature and phosphor self-heating increases the temperature of the phosphor layer. Photoluminescence saturation is not appreciable in the plot since YAG phosphors have a short decay time and the irradiance has been kept at low levels to avoid irreversible damage to the phosphor template.

#### 3.2.2. Reliability Results

The first reliability analysis was carried out by means of a step–stress study; in this test the same phosphor layer was subjected to cumulative stress tests, each characterized by a fixed duration (1 h) and a storage temperature which was increased test after test from 150 to 550 °C, with a step of 50 °C. At the end of each single test the samples were measured at room temperature by means of the aforementioned photoluminescence setup. 

**Figure 6:**
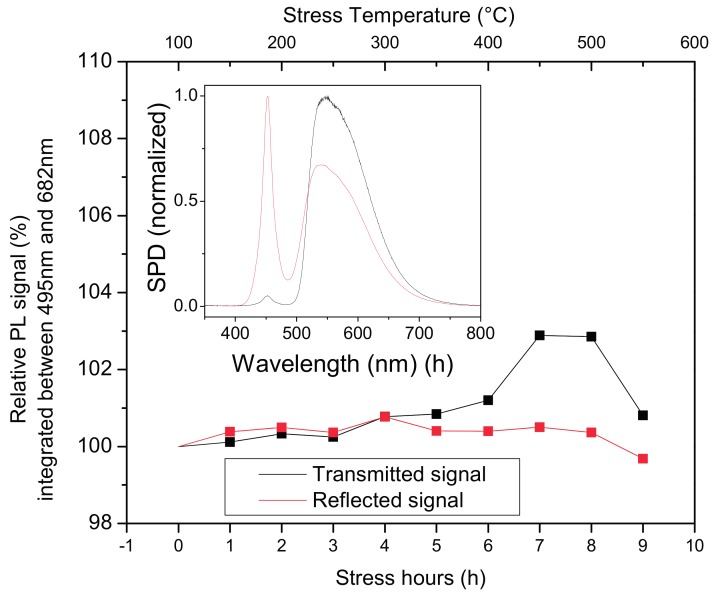
Photoluminescence signal of the YAG:Ce phosphor during the step–stress analysis. Inset: normalized spectrum of the transmitted and reflected signal.

The results indicate that after 1 h of treatment at 550 °C, the phosphors were quite stable, with a loss of less than 1% with respect to the initial value. The inset plot in Figure 6 compares the spectra of the reflected and transmitted radiation; it can be observed that nearly all the LED radiation is absorbed from the phosphor, thus nearly no 450 nm spectrum is present in the transmitted radiation. Nevertheless a high amount of the LED radiation is backscattered and reflected from the phosphor sample. 

Considering the stability of the phosphors discussed above, the reliability test stress temperatures were selected at the maximum values of the step stress test: 500 °C, 525 °C and 550 °C. Reliability test were carried out on different phosphor templates produced at the same conditions of the previous step–stress-tested phosphor. Each sample was subjected to a single temperature storage test for a total of up to 1300 h, the storage test was interrupted for room temperature photoluminescence measurements at predetermined time intervals.

**Figure 7:**
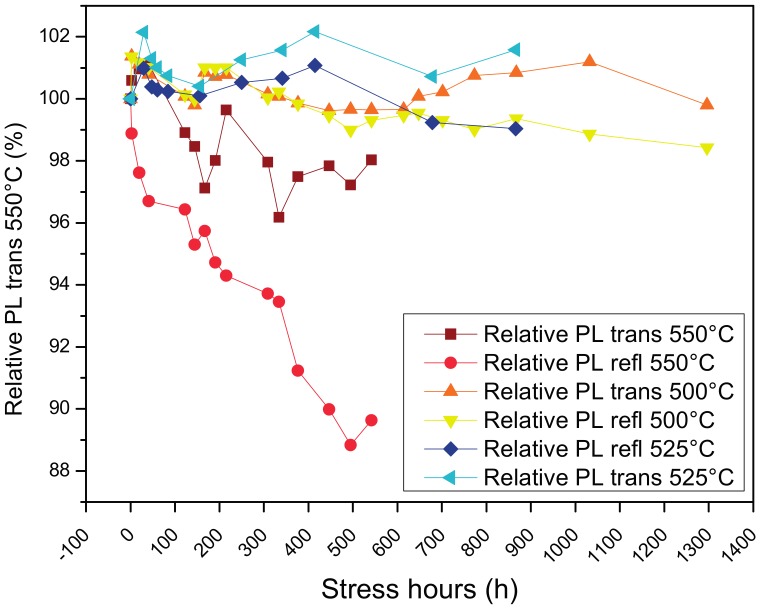
Phosphor photoluminescence during thermal ageing at different temperatures.

Phosphor layers subjected to thermal stress at 500 °C and 550 °C confirmed the good stability (Figure 7) seen in the step–stress analysis; on the other hand, at 550 °C it was possible to notice a certain degradation of the conversion efficiency, which after approximately 500 h showed a degradation of 3% and 10% for transmitted and reflected photoluminescence, respectively. The greater degradation of the reflected measurements can possibly be related to the reduction of efficiency; the first phosphor layers (responsible for the greater part of the reflected photoluminescence) are converting a reduced amount of radiation, thus allowing more, scattered, unconverted blue light to pass through and excite the phosphors on the deeper layers (resulting in a greater contribution to transmitted photoluminescence).

## 4. First Approach: Transmission

The phosphor deposition method implemented in the previous section was found to offer a too large degree of freedom in terms of uniformity and thickness to provide several samples with the same characteristics to be used in the transmission approach, therefore, the remote phosphors of choice were based on commercial templates. The remote phosphor structure used for the development of the LARP transmissive tests was a phosphor blend with YAG as the main component, encapsulated in a silicone matrix, with the phosphor layered on a glass carrier together with a diffuser film. The phosphor template structure was provided as is from a commercial manufacturer. Although this solution was not able to sustain the maximum local irradiance achievable from the laser beam, it allowed very good results in terms of the uniformity of the beam and constant phosphor thickness over the entire sample area. To avoid the direct reflection of the projected laser beam from the air/glass transition, the phosphor layer was placed in the direction of the incoming beam.

**Figure 8:**
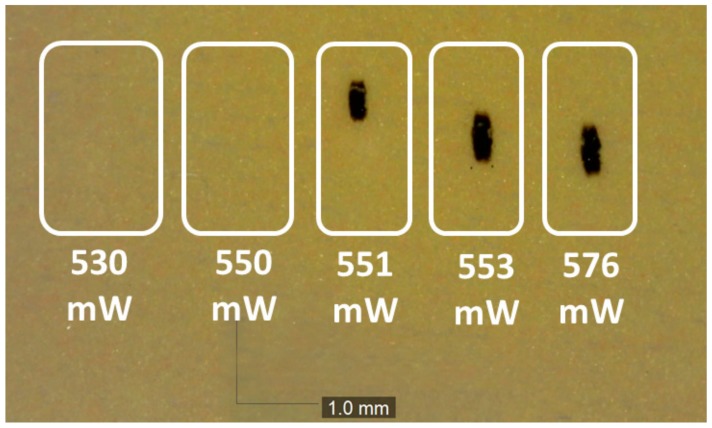
Phosphor catastrophic degradation as a function of incident power.

To assess the maximum optical power density sustainable from the phosphor sample, a simple test based on fixed time laser pulses was carried out. The laser radiation (450 nm from the same laser samples used in this work) was collimated onto the phosphor template, then a series of 10 s long pulses were applied to the phosphor at different optical powers in order to assess the maximum sustainable optical power density. The phosphor template was placed vertically on free air at an ambient temperature of 25 °C. The results are reported in Figure 8. A clear, sharp transition between the sustainable and unsustainable laser radiation was detected, possibly related to the positive feedback thermal effect of the phosphor thermal quenching. It was evaluated that the maximum sustainable optical power was 550 mW over an area of 0.1145 mm^2^, corresponding to an irradiance of 4.8 W/mm^2^, at an LD current of 627 mA. To avoid catastrophic phosphor degradation, a value of 400 mW over an area of 0.1 mm^2^ (4 W/mm^2^), corresponding to a LD current of 499 mA, was selected to be used in the following tests.

### 4.1. Structure Design and Experimental Details

**Figure 9:**
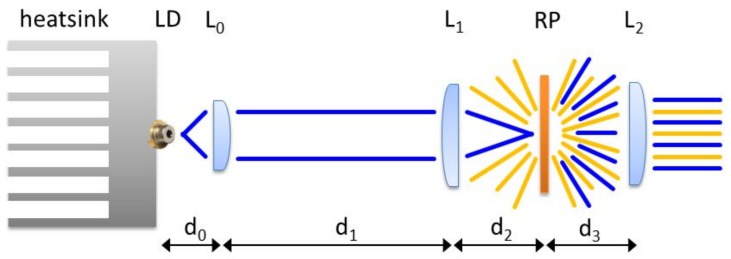
Schematic structure of the transmitted LARP setup.

**Figure 10:**
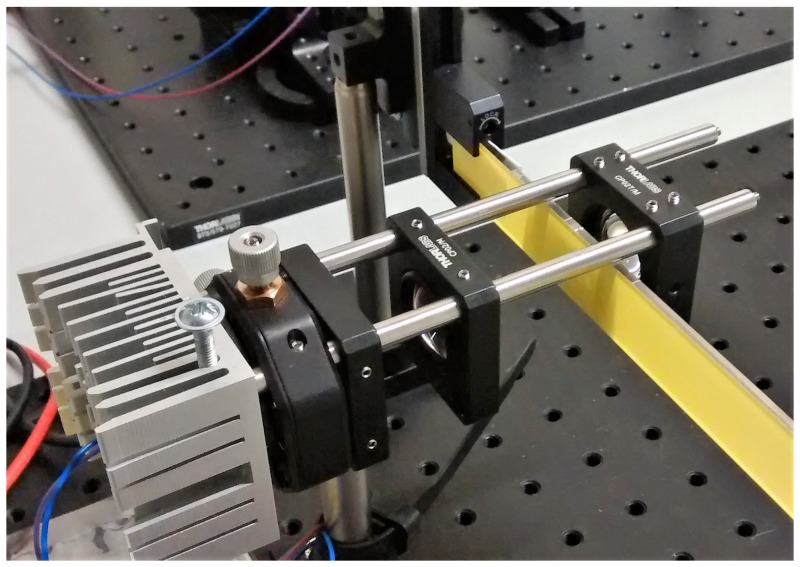
Measurement setup of the transmission LARP system.

The structure of the transmission LARP system (see the sketch in Figure 9 and the final system in Figure 10) is quite simple: the laser diode is thermally connected to an aluminum heatsink; a collimating lens L_0_ is placed on the optical axis at a distance d_0_; at d_1_ we find the spherical focal lens which focalizes the laser beam on the remote phosphor (RP) layer at a distance d_2_. The resulting laser and phosphor light is then collimated by a spherical lens L_2_ placed at a distance d_3_.

Considering that the laser diode has a parallel and orthogonal beam divergence respectively of θ_‖_ = 7° and $\theta_{⊥}$ = 23°, and that the aspheric collimating lens L_0_ has a focal length of 2.76 mm and NA = 0.52, it is possible to calculate:

$$2r_{∥}=2f\tan\theta_{∥}=0.678\;\mathrm{mm}$$

$$2r_{⊥}=2f\tan\theta_{⊥}=2.343\;\mathrm{mm}$$

where 2r_‖_ is the collimated beam diameter in the parallel direction, whereas 2$r_{⊥}$ is the collimated beam diameter in the orthogonal direction. 

The collimated beam then reaches lens L_1_, which focuses the monochromatic radiation on the phosphor layer. L_1_ has a focal length f_1_ = 16 mm and a diameter of 25.4 mm. By varying the distance between the phosphor and L_1_ it is possible to modify the size of the laser spot on the phosphor, the chosen distance for the proposed design is d_2_ = 17 mm.

The projection lens was designed to allow a good collection of emitted light and also a small divergence angle. L_2_ has a focal length and diameter both of 25.4 mm. The distance d_3_ from the phosphor surface is 8 mm.

From the measurement of the optical beam characteristics, the emitted beam from the optical structure has been projected over a white reference screen, placed at 1320 mm from the L_2_ lens, where the intensity and RGB coordinates were measured by means of a calibrated RGB charge-coupled device (CCD) camera. The single color channels of the sensor were plotted to identify the different color angular distributions of the projected light beam.

To measure the total flux emitted from the light source, the entire setup was placed inside a 1.65 m Labsphere LMS-650, equipped with a spectrometer and calibrated both in absolute luminous flux and absorption from the source itself. Several absolute spectrum and luminous flux measurements were therefore carried out at different LD operating currents.

### 4.2. Measurement Results

**Figure 11:**
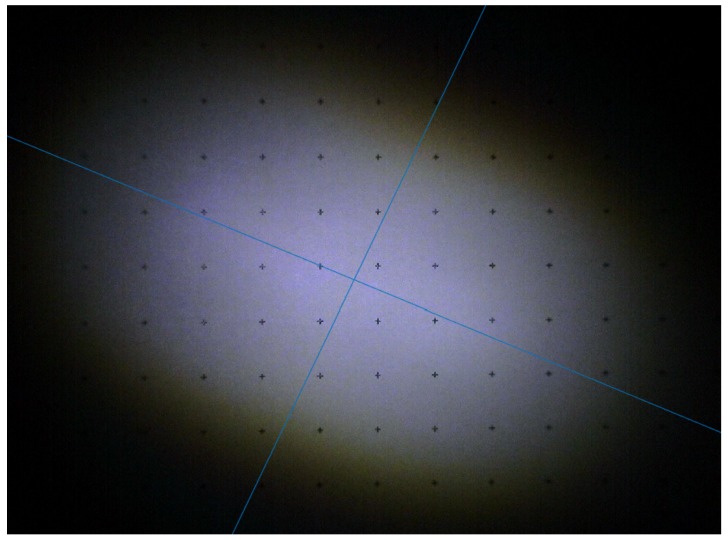
Target spot of the transmission LARP setup at a distance of 1320 mm from the light source.

The measurement results are presented here for the transmission mode projection setup. In Figure 11 it is possible to see the elliptical spot of the laser projector; considering that the dot grid has a step size of 10 mm, the size of the spot at a distance of 1320 mm is approximately 135 × 95 mm^2^. The spot size allows the evaluation of the beam full width at half maximum (FWHM) divergence, which is 2.7° for the main axis and 2.1° for the transverse axis. 

**Figure 12:**
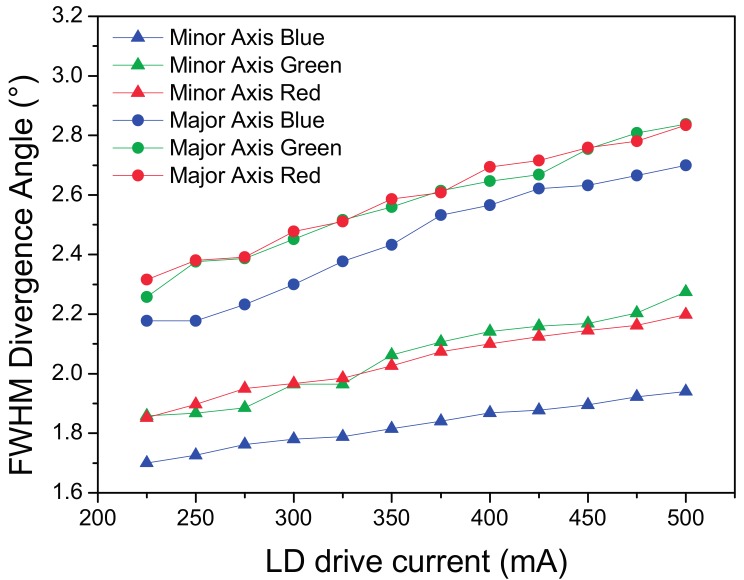
RGB FWHM divergence angle analysis for the transmission LARP setup on the major axis.

In Figure 12 it is possible to see the R, G and B FWHM divergence angles at different currents. 

By comparing the different color channels on the same plot, it is possible to notice that, whereas for the main axis the distribution has the same size between the different colors, on the minor axis the blue emission is notably smaller, thus inducing a certain yellow color shift on the extreme border of the projection spot, as visible in Figure 11. 

By analyzing the plot reported in Figure 12, a clear increase in the spot divergence occurs as the LD current increases, this mechanism is related to the confinement of the radiation emitted from the laser facet, as the current increases the optical and current confinement under the laser ridge becomes less effective, thus leading to the increase of the light emitting surface and the corresponding source étendue.

**Figure 13:**
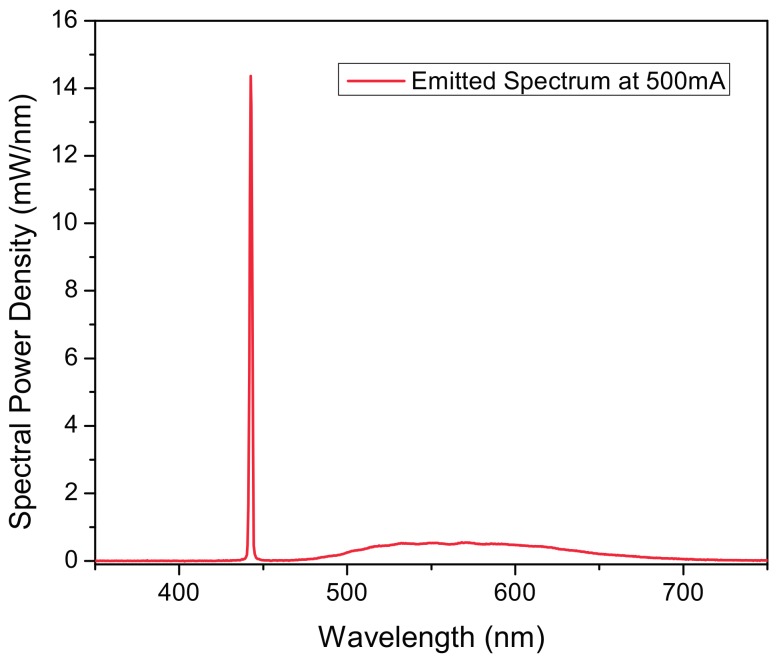
Transmission LARP-emitted output spectrum.

Absolute spectral results from the integrated sphere measurements are reported in Figure 13, compared to the typical spectrum of a phosphor converted LED, the spectral power density of the blue peak is much higher (31 times the maximum spectral power density of the phosphor photoluminescence). Colorimetric characteristics of the emitted light show a correlated color temperature (CCT) of 8429 K, color rendering index 75 and chromatic coordinates of x = 0.3023, y = 0.2711 at an LD current of 500 mA. These values are related to phosphor layer composition, mixture and thickness. Color temperature can be reduced by increasing the thickness of the phosphor layer, however, since a yellow–green YAG phosphor is used, the chromatic balance and color rendering would be too far away from the black body locus and too low, respectively, to be considered white light. Therefore, the achieved values are considered a fair approximation of a white light source.

**Figure 14:**
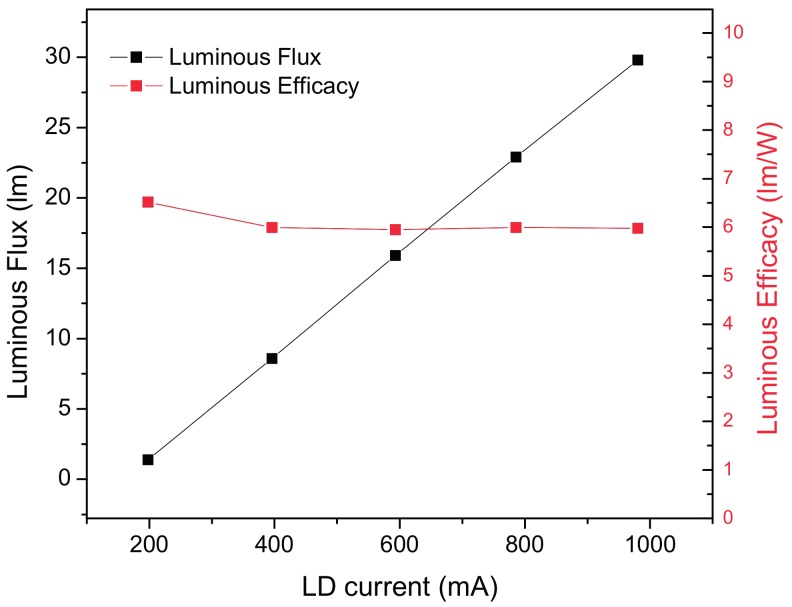
Luminous flux and luminous efficacy of the transmission LARP system.

The luminous flux vs. LD current plot is reported in Figure 14. Although the absolute value of 12.5 lm at 500 mA indicates a luminous efficiency of just 5.95 lm/W, it is possible to observe an excellent linearity of the luminous flux with respect to the driving current of the LD, without any sensible decrease in efficiency. The low value of the efficiency, related to the maximum theoretical value of 50 lm/W (for this combination of laser diode and phosphor structure), can be explained by: (a) the low current driving of the LD (to avoid phosphor degradation), representing a 20% reduction; (b) the bilateral emission of the phosphor, representing a further 50% reduction; (c) the optical loss related to the lens acceptance angle, representing the remaining 70% of reduction, to achieve the total 88% of lumen loss. This mainly related to: (1) optical losses in the coupling of the collimating lens with the laser diode; and (2) the fact that the phosphor is emitting in every direction and optical losses are important in the coupling of the focalizing lens with the emitting phosphor layer.

The CCT was found to increase as the LD driving current increases, this behavior is most likely related to a YAG:Ce reduced blue light absorption as temperature increases, rather than saturation [9]. The CCT shifts from 8050 K at a LD current of 250 mA to 8429 K at 500 mA.

## 5. Second Approach: Reflection

A reflective projection system is structurally similar to the transmissive setup, but the phosphor layer is deposited on a reflective carrier, instead of a transparent one. The optical structure of the projector is therefore slightly complicated by the fact that the lenses and reflectors are not placed on the same optical axis, but require specific positioning. We here evaluated two approaches, one using a tilted phosphor target and a second one using a perpendicular phosphor target and a parabolic mirror. The reflective phosphor carrier in the proposed experiments is a glass substrate with a dielectric mirror on the top surface; the phosphors were deposited directly on the reflective surface. 

### 5.1. Setup with Reflection on a Tilted Phosphor Target (REFL1)

**Figure 15:**
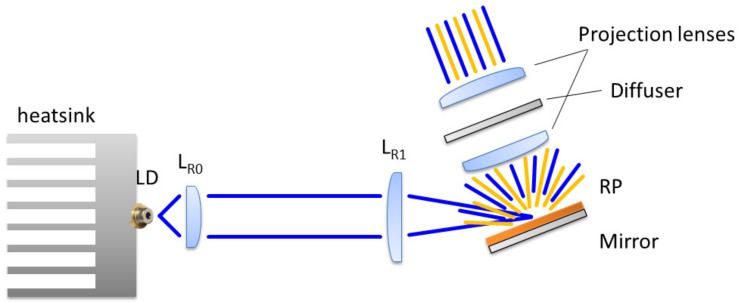
Schematic structure of the reflected LARP setup nr. 1.

The projection setup is presented in Figure 15. It is possible to notice that in order to guarantee the maximum amount of light on the projection lenses, the incoming LD blue beam is nearly at a tangent to the phosphor layer. Although this solution allows a good efficiency, it has the disadvantage of a deformation and an increase of size of the blue laser spot on the phosphor layer. This results in a greater asymmetry of the projected spot. To partially compensate for this phenomenon and improve color uniformity, an engineered round diffuser was placed between the two projection lenses.

### 5.2. Setup with Perpendicular Reflection and Parabolic Mirror (REFL2)

**Figure 16:**
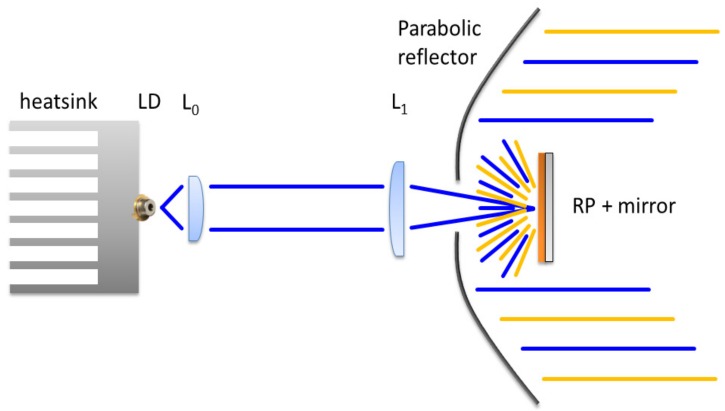
Schematic structure of the reflected LARP setup nr. 2.

A second approach, presented in Figure 16, is based on a parabolic reflector (PR). The design of the projection structure is based on the laser beam passing through an aperture on the PR, which excites the remote phosphor (RP) layer deposited on a specular reflective carrier. The phosphor sample is positioned on the focal point of the PR to obtain a collimated beam. The parabolic reflector has a diameter of 90 mm, a height of 40 mm and a focal distance of 12.66 mm.

### 5.3. Experimental Details

The YAG phosphors were deposited with the same drop casting method described in the reliability section, but the carrier is a 12.7 mm broadband dielectric mirror with a reflectance above 99% in the range between 400 nm and 750 nm.

To measure the performances of the two described LARP systems, a specific setup was adopted. A reference white panel decorated with 10 mm spaced reference dots was placed at a distance of 330 mm from the LARP source. 

### 5.4. Results

**Figure 17:**
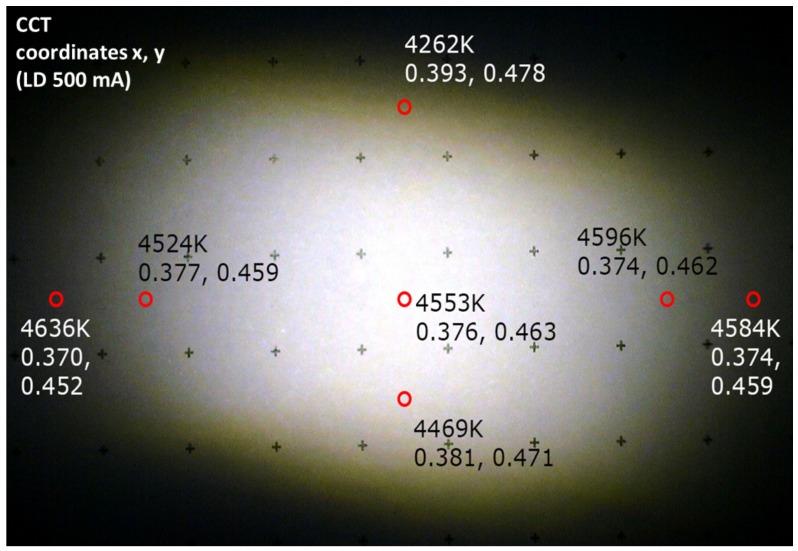
Target spot of the reflective LARP setup REFL1 (tilted reflector) at a distance of 330 mm from the light source.

Figure 17 reports the light pattern emitted from the system based on a tilted reflector (REFL1, Figure 12) as projected onto a reference white panel placed at a distance of 330 mm. The beam has a FWHM at the main axis of 57.3 mm, whereas the minor axis has a length of 53.2 mm, resulting in a beam angle of 5.94°. The CCT of the emitted beam is sensibly lower than that in the transmission, and is approximately equal to 4500 K, with good color over angle uniformity (see Figure 14 for details). 

**Figure 18:**
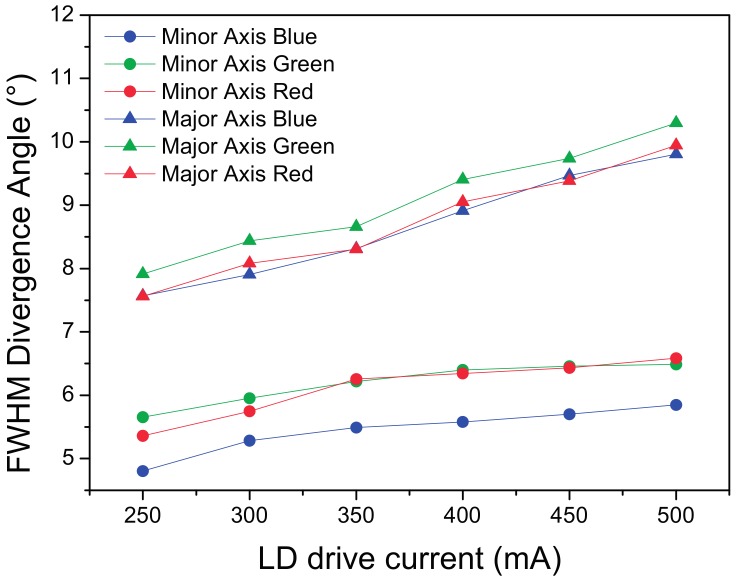
RGB FWHM divergence angle analysis for the reflective LARP setup nr. 1.

The size of the beam, in particular for the main axis, increases with the driving current. Figure 18 reports the R, G and B plot on the projection surface.

**Figure 19:**
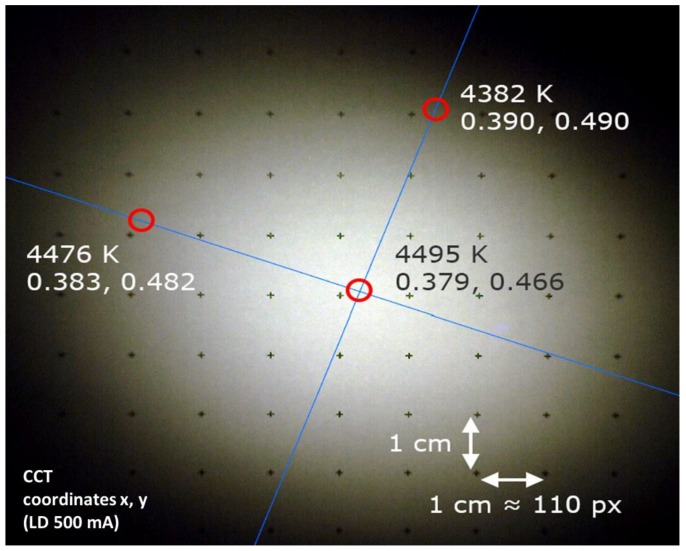
Target spot of the reflective LARP setup nr. 2 at a distance of 330 mm from the light source.

**Figure 20:**
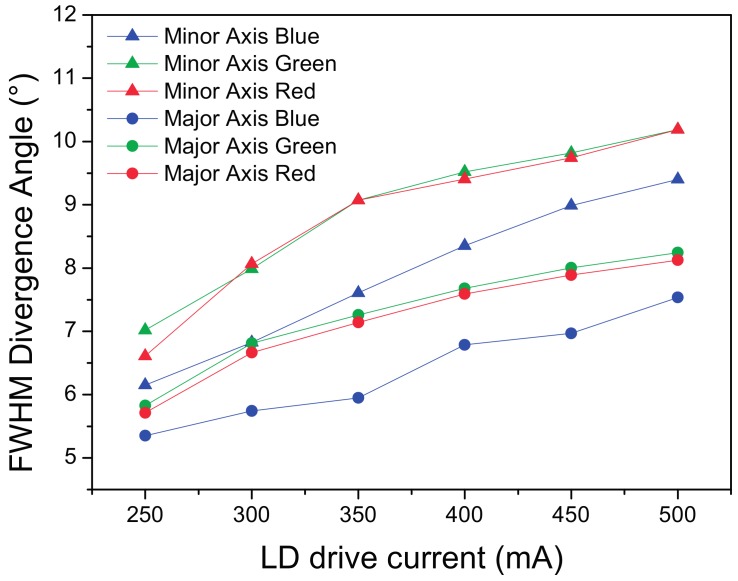
RGB FWHM divergence angle analysis for the reflective LARP setup nr. 2.

Similar results were obtained for the REFL2 setup based on the parabolic reflector (see Figure 19 and Figure 20). The FWHM along the two axes are 67 and 54 mm, respectively, corresponding to 9.8° and 6°. The CCT also has a value of 4495 K in the center, and reaches 4382 K at the extreme borders of the projected spot. The CCT decrease from center to border is related to the smaller size of the blue spot with respect to the yellow–green phosphor beam.

**Figure 21:**
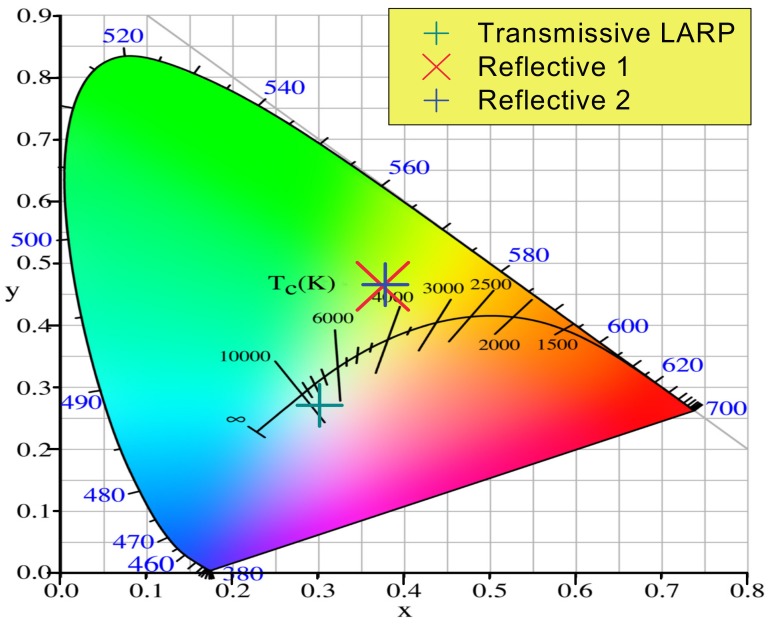
Chromatic properties of the beam emitted from the three LARP setups.

The output of the three analyzed systems was also measured by means of an Ocean Optics USB4000 CCD spectrometer equipped with a cosine corrector. The position of the white point on the chromatic diagram is reported in Figure 21. For all the analyzed samples, the white point is quite far from the black body locus, by adjusting the phosphor thickness it is possible to tune the CCT in a straight line between the 450 nm monochromatic coordinates (x = 0.16; y = 0.02) and the coordinate of the phosphor spectrum (x = 0.426; y = 0.548). All the analyzed test structures present a smaller divergence for the blue component with respect to the phosphor converted radiation. This behavior is related to the different light generation processes for the two components: blue light is simply scattered from the phosphor particles without being absorbed, whereas the yellow/green component is emitted from the phosphors as a consequence of photoluminescence with a quasi-Lambertian emission pattern, the ratio between the two physical mechanisms leads to divergence disuniformities. By tuning the particle size, thickness, structure and density of the phosphors it is possible to control the ratio between the scattering and photoluminescence emission angles.

The results from the luminous flux and relative luminous efficacy measured on the LARP reflective setups show a substantial improvement with respect to the transmissive setup. The measurements performed at an LD current of 500 mA corresponding to an electrical power of 2.083 W show a total luminous flux of 25.2 lm and 38.9 lm for LARP setup REFL1 and REFL2, respectively; the luminous efficacy is 12 lm/W and 18.7 lm/W, respectively.

## 6. Discussion and Conclusions

We presented an extensive analysis of the issues related to the design of laser-based light sources for general lighting. The study is based on both design considerations and experimental tests aimed at evaluating issues such as phosphor degradation, phosphor thermal quenching, lifetime and lamp layout. This latter aspect was investigated by evaluating different approaches/lamp layouts, including total transmission, total reflection on tilted target, and total perpendicular reflection. 

Laser lighting systems are interesting for general lighting, thanks to the absence of efficiency droop and several orders of magnitude lower étendue; they may present some clear advantages with respect to LED-based light systems. LARP lighting also present some challenges, which we tried to address in this work: (i) extremely high irradiance on the phosphor layer possibly inducing faster degradation of the luminescent material; (ii) the complex optical structure of the lighting system; and (iii) color mixing and beam shape.

The maximum sustainable irradiance was studied on YAG phosphor with a silicone binder, indicating a critical value of 4.8 W/mm^2^. The photoluminescence lifetime was found to be in the order of 72 ns, and the reported value was found to gradually increase with temperature, by 4% from 25 to 200 °C.

Both the thermal reversible and irreversible efficiency decrease were analyzed, and the results indicate that thermal quenching is a clear issue at temperatures above 200 °C, which can easily be reached during laser excitation. The results reported in this work indicate that a good stability of the YAG:Ce phosphor is achievable up to an operating temperature of around 525 °C, above which value a certain degradation of the conversion efficiency starts to appear. 

Both transmission and reflection LARP setups were designed and tested in this work. For the transmission setup, a really low divergence was achieved (2°) by means of a 25.4 mm lens; for comparison, LED systems with similar input optical power require lenses with a diameter in the order of 185 mm [21]. This system also suffers from major issues: (i) the optical efficiency is very low (10%); (ii) the color mixing is not perfect, and the light source has a high CCT. The authors consider that these disadvantages can be minimized by means of ad-hoc designed lenses and optical systems and by using more uniform, multi-wavelength phosphor layers. For the reflective setups, both lenses and parabolic reflectors were tested, and although the beam divergence of around 5° is sensibly higher with respect to the transmissive setup, uniformity and efficiency were substantially improved.

Comparing the luminous efficiencies, we report 6 lm/W, 12 lm/W and 18.7 lm/W for the transmissive, reflective 1 and reflective 2 LARP setups, respectively. In all the analyzed setups, a gradual increase of the emitted beam divergence was found as the LD current increases, possibly as a function of reduced carrier and optical confinement in the LD active region. The parabolic setup at this stage is the most promising solution, for its simplicity and efficiency.
